# Biocompatible quaternized chitosan-based nanocomposite hydrogels with antibacterial and rapid hemostatic properties

**DOI:** 10.1039/d5ra03440j

**Published:** 2025-08-26

**Authors:** Juanni Zhang, Farhan Mohd Said, Ruixue Lv, Nur Fathin Shamirah Daud, Zhanxin Jing

**Affiliations:** a Faculty of Chemical and Process Engineering Technology, Universiti Malaysia Pahang Al-Sultan Abdullah Lebuh Persiaran Tun Khalil Yaakob 26300 Kuantan Pahang Malaysia farhan@umpsa.edu.my; b College of Chemistry and Environment, Guangdong Ocean University 524088 Zhanjiang Guangdong China

## Abstract

In this study, we developed a quaternized chitosan-based nanocomposite hydrogel by combining dual-network and nanocomposite technology. Firstly, quaternized chitosan (QCS) and chitin nanowhiskers (ChWs) were synthesized and characterized. The quaternized chitosan-based nanocomposite hydrogels were constructed by the radical polymerization of acrylic acid (AA) and acrylamide (AM) and the subsequent cooling process in the presence of QCS, ChWs, and Zn^2+^. The chemical structure and morphology of the synthesized hydrogels were analyzed using FT-IR and SEM, revealing that the nanocomposite hydrogels have a remarkable three-dimensional network structure. The effects of QCS, ChWs, and Zn^2+^ content on the hydrogel's physical and biological properties were systematically investigated. The swelling behavior, mechanical strength, and antibacterial performance of quaternized chitosan-based nanocomposite hydrogels can be effectively modulated by varying their composition. An increase in QCS content led to a notable enhancement in mechanical properties. Specifically, the hydrogel containing 25% QCS exhibited a tensile strength of 391.9 kPa and an elongation at break of 495%. The increased QCS and Zn^2+^ contents significantly improved the antibacterial properties of the nanocomposite hydrogels. The antibacterial rate against *E. coli* and *S. aureus* could reach up to 99%. Furthermore, the QCS-based nanocomposite hydrogels demonstrated good biocompatibility and rapid hemostatic ability. We expect that this simple strategy combining nanocomposite technology and dual-network technology will enrich the avenues for exploring hydrogels with excellent mechanical strength, antibacterial activity, and hemostatic performance for biomedical applications such as wound management, hemostatic materials, and infection control.

## Abstract 2

In this study, we developed a quaternized chitosan-based nanocomposite hydrogel by combining dual-network and nanocomposite technology.

## Graphical abstract

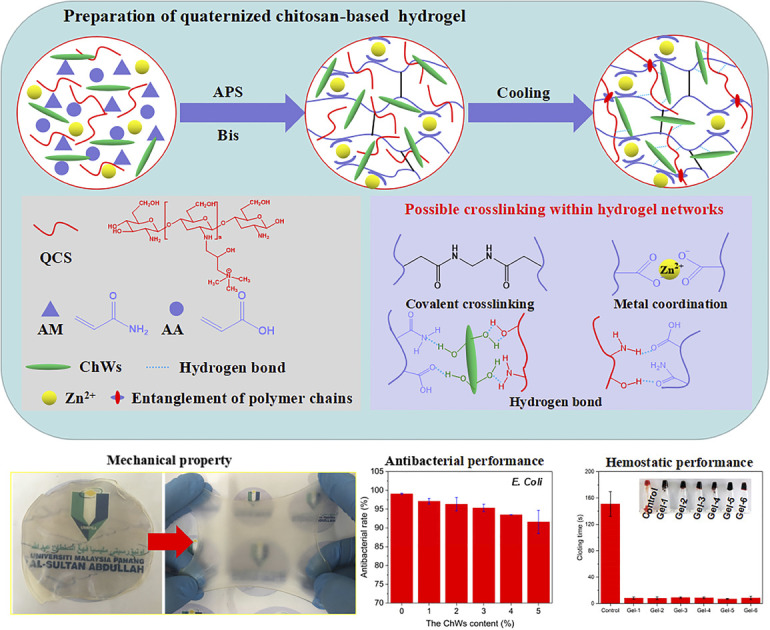

## 1. Introduction

Chitin, a linear polysaccharide consisting of *N*-acetylglucosamine linked through β-1, 4-glucoside bonds, is widely distributed in the exoskeletons of crustaceans and insects as well as in the cell walls of fungi.^1^ Based on its crystal structure, chitin can be classified into three distinct types: α-, β-, and γ-chitin.^2^ Among these, α-chitin is the most common type with tightly packed molecular chains exhibiting stable chemical properties. At present, chitin has attracted considerable attention from researchers because of its remarkable biodegradability, biocompatibility, renewability, and non-toxicity.^3^ Chitin nanowhiskers (ChWs) are nano-scale products derived from chitin, usually in rod or spindle shapes.^4^ They generally have lengths ranging from tens to hundreds of nanometers and diameters from a few to several tens of nanometers. These nanowhiskers exhibit higher crystallinity than bulk chitin,^5^ resulting in significantly enhanced mechanical properties such as strength and modulus.^6^ Additionally, ChWs also exhibit superior mechanical attributes, chemical surface reactivity, a high specific surface area, biodegradability, biocompatibility, and antibacterial performance.^7^ The physical and chemical properties of ChWs make them highly promising for various applications. Therefore, ChWs have been used in many fields, such as drug delivery,^8^ tissue engineering,^9^ environmental remediation,^10^ and the food industry.^11,12^

Chitosan, a prominent derivative of chitin, is produced through the deacetylation process of chitin, resulting in the removal of *N*-acetyl groups.^13^ Chitin is classified as chitosan when its degree of deacetylation exceeds 50%. The degree of deacetylation plays a crucial role in determining the characteristics of chitosan, including solubility, antimicrobial activity, and chemical reactivity.^14,15^ Highly deacetylated chitosan exhibits antimicrobial and chemical properties due to its abundant amino groups (–NH_2_), so chitosan-based materials have diverse applications in biomedicine,^16^ food science,^17^ environment,^18^ and the cosmetics industry.^19^ Chitosan hydrogels are an attractive class of chitosan-based materials due to their biodegradation, biocompatibility, and antibacterial properties. However, some deficiencies in the properties of chitosan-based hydrogels, such as suboptimal mechanical properties and antibacterial properties, have limited their application in many fields. Quaternization of chitosan is considered to be an effective method to improve the antibacterial properties of chitosan-based hydrogels.^20^ Fan *et al.*^20^ synthesized hydrogels composed of quaternary ammonium chitosan (HACC), polyvinyl alcohol (PVA), and polyethylene oxide (PEO), and found that the hydrogel containing HACC showed significant antibacterial activity against *S. aureus* and *E. coli*. A self-adhesive and self-healing hydrogel based on quaternary ammonium chitosan and silk fibroin was reported by Guo *et al.*^21^ The results also demonstrated that quaternary ammonium chitosan was incorporated into the hydrogel to enhance its antibacterial ability.

At present, many methods, such as nanocomposite technology,^22,23^ double network technology,^24,25^ and supramolecular technology,^26^ have been used to improve the mechanical properties of hydrogel materials. In the study by Chang *et al.*,^22^ nanohydroxyapatite (nHAp) was incorporated into chitin hydrogels. The results demonstrated a significant improvement in the mechanical properties of the chitin/nHAp hybrid hydrogel, with its compressive strength reaching 274 kPa, approximately 10 times that of the pristine chitin hydrogel, thereby providing strong evidence for the reinforcing effect of nanocomposite technology. Pourjavadi and colleagues successfully fabricated chitosan/polyvinyl alcohol composite hydrogels using a double-network strategy.^24^ Compared to single-network polyvinyl alcohol hydrogels (tensile strength: 1.48 MPa; elongation at break: 136.6%), the mechanical properties of the double-network hydrogel were significantly enhanced, with its tensile strength reaching 11.52 MPa and elongation at break improving to 265.6%. This result indicates that constructing a double-network structure can effectively enhance the mechanical properties of hydrogels. Additionally, Mohamadhoseini *et al.*^27^ used supramolecular technology to fabricate self-healing hydrogels based on host–guest interactions, achieving a maximum modulus of 6500 Pa, further demonstrating the potential of supramolecular technology in enhancing the mechanical properties of hydrogels.

Thus, in this study, we constructed quaternized chitosan-based hydrogels by integrating nanocomposite technology and dual network technology to enhance their mechanical properties while imparting excellent antimicrobial properties. First, QCS and ChWs were prepared and characterized. Afterward, the quaternized chitosan-based nanocomposite hydrogels were synthesized, and their chemical structure and surface morphology were analyzed using FT-IR and SEM. Subsequently, the effects of QCS, ChWs, and Zn^2+^ contents on the swelling, mechanical, and antibacterial properties of the synthesized hydrogels were evaluated. Eventually, their biocompatibility and blood coagulability were analyzed to explore their potential application as hemostatic materials.

## 2. Materials and methods

### 2.1 Materials

Chitin, ammonium persulfate (APS, 98.5%), acrylamide (AM, 99.0%), dimethyl sulfoxide (DMSO), 2, 3-epoxy-propyl trimethyl ammonium chloride (≥95%), zinc nitrate hexahydrate (Zn(NO_3_)_2_·6(H_2_O), 99%), and hexadecyl trimethyl ammonium bromide (CTAB) were all purchased from Shanghai Macklin Biochemical Technology Co., Ltd, China. Chitosan (CS, Biological reagent) was purchased from Sinopharm Chemical Reagent Co., Ltd, China. Acrylic acid (AA, 98%) and *N*,*N'*-methylene bisacrylamide (Bis, 98%) were purchased from J&K Scientific Co., Ltd, China. Other reagents were analytically pure and were not further purified.

### 2.2 Preparation of quaternized chitosan (QCS)

QCS was prepared by the following description.^28,29^ The chitosan was first treated with deacetylation. 10 g of chitosan, 20 g of sodium hydroxide (NaOH), 30 mL of deionized water, and 50 mg of hexadecyl trimethyl ammonium bromide (CTAB) were dissolved in 200 mL of dimethyl sulfoxide (DMSO). The mixture was placed in an ultrasonic cleaner operating at a frequency of 90 Hz and a temperature of 75 °C for 2 h. Subsequently, the mixture was transferred to a water bath at 75 °C for 2 h. After centrifugation, the resulting filter residue was washed with deionized water until neutral. Finally, the collected product was vacuum-dried at 70 °C for 10 h and set aside.

Further, QCS was synthesized by modifying deacetylated chitosan with 2,3-epoxypropyltrimethyl ammonium chloride (GTA). 2.5 g of deacetylated chitosan was added to 100 mL of isopropanol and stirred at 80 °C for 6 h. During this step, the pH was adjusted to a range of 8–9 using a 1% NaOH solution. Next, 20 mL of GTA solution (62.5 mg mL^−1^) was added and maintained at 80 °C for 24 h. Eventually, the mixture was dialyzed for two days to remove any unreacted GTA, and the collected solution was freeze-dried.

### 2.3 Preparation of ChWs

ChWs were prepared according to the previous literature.^4,30^ Initially, 5 g of chitin was placed into 150 mL of 5% KOH solution, and the mixture was brought to a boil and stirred for 6 h. Subsequently, the mixture was stirred at room temperature for 12 h. After filtration, the collected residue was washed with deionized water until neutral. The collected mixture was transferred to a NaAc/HAc buffer solution with a pH of 4, and 150 mL of 1.7% sodium chlorite solution was added and bleached at 80 °C for 2 h. The bleaching process was repeated by filtering and adding fresh sodium chlorite solution to ensure complete decolorization. The collected substance was treated with 5% KOH for 2 days. Eventually, the substance obtained by centrifugation was washed three times with deionized water and dried at 50 °C to obtain purified chitin.

Further, the purified chitin was added to the HCl solution (3 mol L^−1^) according to the ratio of 1 g of purified chitin to 30 mL of HCl solution, and boiled and stirred. After 90 min, 50 mL of deionized water was added, and the mixture was centrifuged at 10 000 rpm for 5 min. The collected precipitate was washed three times to remove the residue of HCl. Finally, the precipitate was collected and resuspended in deionized water, followed by dialysis until the pH reached neutrality. The suspension was adjusted to a pH of 3 and ultrasound for 60 min. Eventually, the suspension was freeze-dried to obtain ChWs.

### 2.4 Preparation of quaternized chitosan-based nanocomposite hydrogels

A specific quantity of ChWs and QCS was added to 10 mL of deionized water and stirred at room temperature for 1 h. Subsequently, 1.4 mL of acrylic acid (AA), 0.6 g of acrylamide (AM), and a variable volume of 0.5 mmol mL^−1^ Zn^2+^ solution were added, and the mixed solution was stirred for 10 min. Afterward, 2 mg of *N*,*N′*-methylene bisacrylamide (Bis) and 20 mg of ammonium persulfate (APS) were added, and followed by stirring for an additional 10 min to ensure thorough incorporation. The prepared solution was transferred into a mold and placed at 60 °C for 12 h. Eventually, the obtained sample was kept at room temperature for 10 h to obtain the final hydrogel. The specific composition of quaternized chitosan-based nanocomposite hydrogels is listed in Table 1.

**Table 1:** The composition of quaternized chitosan-based nanocomposite dual-network hydrogels

Codes	ChWs^a^ (%)	QCS^b^ (%)	Zn^2+^ (mmol)
Gel-1	0	10	0.10
Gel-2	1	10	0.10
Gel-3	2	10	0.10
Gel-4	3	10	0.10
Gel-5	4	10	0.10
Gel-6	5	10	0.10
Gel-7	5	0	0.10
Gel-8	5	5	0.10
Gel-9	5	15	0.10
Gel-10	5	20	0.10
Gel-11	5	25	0.10
Gel-12	5	15	0.00
Gel-13	5	15	0.05
Gel-14	5	15	0.15
Gel-15	5	15	0.20

a Represents the percentage of ChWs in the total weight of AM, AA and QCS.

b Represents the percentage of QCS relative to the total weight of AM and AA.

### 2.5 Characterization

FT-IR spectra of the samples were performed on a Thermo Fisher Nicolet iS50 FT-IR spectrometer within the range of 4000 cm^−1^ – 400 cm^−1^ at a resolution of 4 cm^−1^. X-ray diffraction (XRD, Raguku Ultima IV, Japan) was used to detect the crystallinity structures of the samples in the range of 5°–80° at a speed of 10° min^−1^. The morphology of the sample was observed by a scanning electron microscope (SEM SU8230, Hitachi). For the hydrogel samples, they were prepared by reaching swelling equilibrium in a buffer solution (pH = 7.4, *I* = 0.05) and freeze-drying. Morphology of ChWs was further observed by transmission electron microscopy (TEM). Thermogravimetric analysis (TGA) was conducted under a nitrogen atmosphere with a heating rate of 10 °C min^−1^, raising the temperature from room temperature to 600 °C. The mechanical properties of the rectangular samples (6 × 2 × 20 mm^3^) were examined using a YHS-229WG universal testing machine (Shanghai YiHuan, China) at a tensile speed of 100 mm min^−1^. At least three specimens were measured for each sample. The rheological properties of the hydrogel sample were conducted on a 25 mm parallel plate rheometer (Anton-Paar MCR302) at room temperature by the following description:^31^ (a) a dynamic frequency sweep was performed from 0.1 to 100 rad s^−1^ at a constant strain of 1.0%. (b) alternating step strain tests were conducted at a fixed angular frequency of 10 rad s^−1^ using two protocols: (1) switching the strain amplitude between 1% and 100% every 100 seconds, and (2) progressively increasing the strain amplitude to 100%, 200%, 300%, and 400%, with each step lasting 100 seconds.

### 2.6 Swelling performance test

The swelling performance of the sample was evaluated as follows: dry samples (ranging from 20 to 50 mg) were immersed in 50 mL of buffer solutions (*I* = 0.05) with varying pH values. After soaking for a certain time, the samples were removed from the buffer solutions, and their surface liquid was carefully removed with filter paper and weighed. The swelling rate (g g^−1^) and equilibrium swelling ratio (g g^−1^) of hydrogels were calculated according to the formula, respectively:^32^ Swelling rate (g g^−1^) = (*W*_*t*_ − *W*_o_)/*W*_o_, Equilibrium swelling ratio (g g^−1^) = (*W*_e_ − *W*_o_)/*W*_o_, where *W*_o_ represents the original dry weight of the hydrogel, *W*_*t*_ represents the swollen weight of the sample at time *t*, *W*_e_ represents the swollen weight of the sample achieving equilibrium swelling. Each set was tested in triplicate.

### 2.7 Hemolysis test

The hemocompatibility of the hydrogel was evaluated by a hemolysis assay.^33,34^ Firstly, a red blood cell (RBC) suspension was prepared by centrifuging citrated rabbit whole blood at 3000 rpm for 5 minutes at 4 °C. The collected RBC suspension was then resuspended in PBS solution to obtain a 2.5% RBC/PBS solution. Next, 2 mL of the RBC suspension was added to the centrifuge tube containing 5 mg of dried powder sample, and incubated at 37 °C in a shaker for 1 h. Afterward, the mixtures were centrifuged at 3000 rpm for 5 min, and the OD value of the supernatant was measured using a microplate reader (BioTek Instruments, Inc., Winooski, USA) at 540 nm. TritonX-100 solution and PBS were used as the positive and negative controls, respectively. The hemolysis ratio of the hydrogel was calculated *via* the following formula: Hemolysis ratio (%) = (OD_Ex_ − OD_NC_)/(OD_PC_ − OD_NC_) × 100%, where OD_Ex_ represents the OD value of the experimental group; OD_NC_ denotes the OD value of the negative control, and OD_PC_ refers to the OD value of the positive control. Each experimental set was assessed in triplicate.

### 2.8 Cytotoxicity

Cytotoxicity of quaternized chitosan-based nanocomposite hydrogels was evaluated by MTT assay and live/dead staining.^35^ Firstly, the hydrogel samples sterilized by UV irradiation were incubated in Dulbecco's Modified Eagle's Medium (DMEM) supplemented with 10% fetal bovine serum (FBS) at 37 °C for 24 h. The original extract was obtained by filtering, and diluted to different concentrations (250, 500, and 1000 μg mL^−1^). Afterward, 100 μL of the L929 cell suspension was seeded into a 96-well plate and incubated at 37 °C with 5% CO_2_ for 24 h. After removing the medium, 100 μL of the extract was added, and cultured for different durations (24 h, 48 h, and 72 h). Then, 20 μL of 0.5 mg mL^−1^ MTT solution (3-(4,5-dimethylthiazol-2-yl)-2,5-diphenyltetrazolium bromide) was added and cultured at 37 °C for 1 h. The medium was removed, and 150 μL of dimethyl sulfoxide (DMSO) was added to dissolve the formazan crystals. The OD value of the solution was measured at 570 nm using a microplate reader. The cell viability (%) was calculated as follows: Cell viability (%) = OD_sample_/OD_control_ × 100%, where OD_sample_ represents the OD value of the sample group, and OD_control_ represents the OD value of the control group. For each condition, the MTT assay was repeated three times, and the results were averaged. Briefly, live/dead staining assay was performed by the following description: The cells were first washed 1–2 times with PBS (Gibco) to remove the residual medium. Subsequently, 100 μL Calcin-AM solution was added and incubated in the dark for 20 min. After removing the Calcein-AM solution, 100 μL of PI diluent was added and incubated for 5 min under the same condition. Finally, the cells were observed by fluorescence microscopy (Olympus, Tokyo, Japan).

### 2.9 Antibacterial test

The antibacterial properties of the hydrogels against *S. aureus* and *E. coli* were assessed through a combination of qualitative analysis (agar diffusion method) and quantitative analysis (plate counting method). The agar diffusion method was performed as described. 100 μL of activated bacterial solution was inoculated into 100 mL sterile agar solution and cultured for 12 h to obtain the original bacterial solution. The original bacterial solution was then subjected to gradient dilution to prepare a bacterial suspension, with *E. coli* being diluted 10^3^-fold and *S. aureus* 10^4^-fold. Subsequently, 100 μL of the bacterial suspension was uniformly spread on a sterile solid agar medium. Dried samples sterilized by UV light were placed on an agar medium inoculated with bacterial suspension, and cultured in an incubator at 37 °C for 24 h, during which the formation and size of the inhibition zone around the sample were observed and photographed.

The plate counting method was performed by the following description. A 5 mL bacterial suspension (*S. aureus* 2.45 × 10^7^ CFU mL^−1^, *E. coli* 1.49 × 10^7^ CFU mL^−1^) was added to the centrifuge tube containing dried sample (∼45–50 mg), and incubated at 37 °C and 100 rpm for 12 h. Subsequently, the bacterial suspension was diluted in a gradient manner, including a dilution factor of 10^5^ for *E. coli* suspension and 10^4^ for *S. aureus* suspension. 100 μL of the diluted bacterial suspensions were evenly spread onto the surface of sterile solid agar medium, and incubated at 37 °C for 24 h. Eventually, the number of colonies on the solid agar medium was observed and counted. The bacterial suspension without hydrogel samples served as the control group. The antibacterial rate (%) was calculated as follows: Antibacterial rate (%) = (*N*_c_ − *N*_e_)/*N*_c_ × 100%, where *N*_c_ is the number of colonies in the control group, and *N*_e_ is the number of colonies in the experimental group containing hydrogel samples. The independent experiment was performed in triplicate, and the antibacterial rate was calculated as the average value with standard deviation (mean ± SD).

### 2.10 Blood coagulation test

#### 2.10.1 Whole blood clotting time (BCT) test^36,37^

20 mg of the dried powder sample was added to a 1.5 mL centrifuge tube and preheated at 37 °C for 3 min. Afterward, 100 μL of preheated rabbit whole blood was added, followed by the addition of 10 μL of 0.2 M CaCl_2_ solution. The mixture was gently stirred and incubated at 37 °C until blood flow was completely nonflowing, and the time recorded for this process was a BCT. Rabbit whole blood without hydrogel served as the control group.

#### 2.10.2 Whole blood clotting index (BCI) test^38^

100 μL of preheated rabbit whole blood was added to the preheated centrifuge tube containing 20 mg of the sample, and 10 μL of 0.2 M CaCl_2_ solution was also added. After the 37 °C incubation for 2 min, 10 mL of deionized water was added. The supernatant was centrifuged at 3000 rpm at 4 °C for 5 min, and its OD value was determined by a microplate reader (BioTek Instruments, Inc., Winooski, USA) at 540 nm. Rabbit whole blood without hydrogel served as the control group. The blood clotting index (BCI, %) was calculated using the following formula:^38^ BCI (%) = OD_e_/OD_c_ × 100%, where OD_e_ represents the OD value of the experimental group; OD_c_ denotes the OD value of the control group. BCT and BCI tests for each hydrogel sample were performed in a minimum of three independent experiments.

## 3. Results and discussions

### 3.1 Synthesis and characterization of quaternized chitosan-based nanocomposite hydrogels

**Scheme 1:**
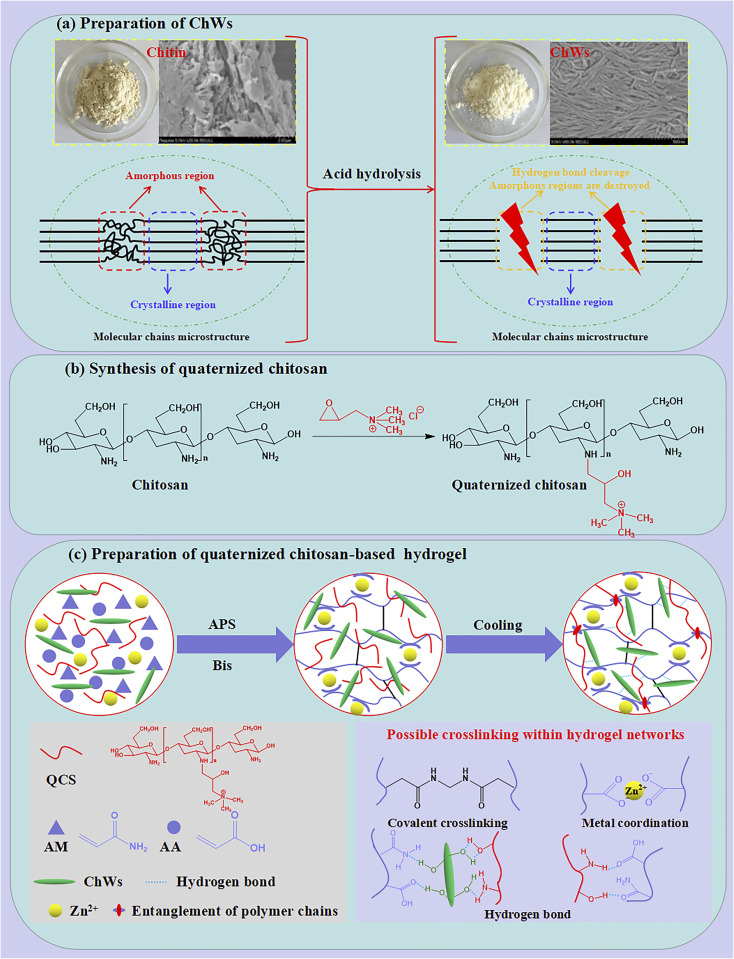
Schematic diagram of the synthesis of quaternized chitosan-based nanocomposite dual-network hydrogels: (a) preparation of ChWs; (b) synthesis of quaternized chitosan (QCS); (c) synthesis of hydrogels and their possible formation mechanism.

**Fig. 1:**
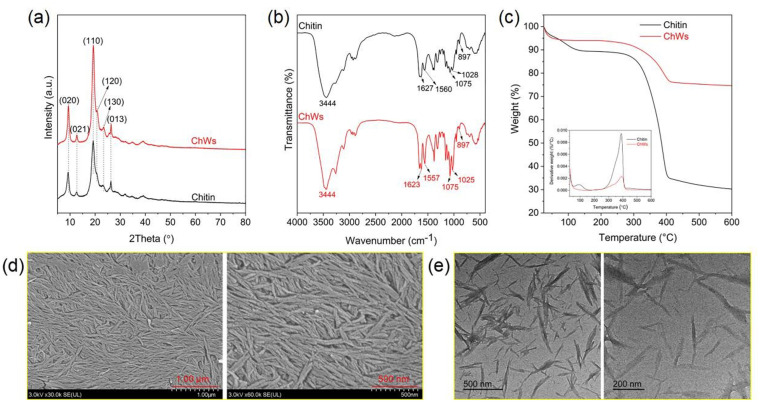
(a–c) XRD patterns (a), FT-IR spectra (b) and TG curves (c) of chitin and ChWs; (d and e) SEM (d) and TEM (e) images of ChWs.

Chitin nanowhiskers (ChWs) were prepared from chitin *via* acid hydrolysis (Scheme 1(a)). During the acid hydrolysis process, the amorphous regions of chitin molecules were selectively removed, leading to the formation of ChWs. Compared with chitin, the ChWs exhibit stronger diffraction peaks at 2*θ* = 9.3, 12.5°, 19.1°, 20.7°, 23.1°, and 26.2° (Fig. 1(a)), corresponding to the (020) (021), (110), (120), (130), and (013) planes, respectively.^39–41^ This indicates that the obtained ChWs have an enhancement in crystallinity, which is due to the selective elimination of the amorphous regions during hydrolysis.^39^ ChWs show a similar FT-IR spectrum to chitin (Fig. 1(b)). Compared with chitin, the O–H stretching vibration peak of ChWs at 3444 cm^−1^ becomes narrower, and the amino (–NH_2_) vibration peak within the 3200–3300 cm^−1^ range sharpens. Furthermore, the amide I band (1623 cm^−1^), the amide II band (1557 cm^−1^), as well as the overlapping C6–O and C=O stretching vibration peak at 1075 cm^−1^, and the C–O–C bridge stretching vibration peak at 1025 cm^−1^ become more distinct and sharper. These variations are presumably attributed to eliminating the amorphous regions of chitin during acid treatment, leading to ChWs with higher crystallinity and a more ordered internal structure. It can be observed from Fig. 1(c) that ChWs display superior thermal stability compared to chitin, mainly due to their higher crystallinity,^42^ making them more resilient to thermal decomposition. Compared to chitin (Fig. S1), the obtained ChWs display a spindle-shaped morphology (Fig. 1(d)), and some ChWs form aggregates or bundle-like structures (Fig. 1(e)). This is mainly due to the removal of amorphous chitin chains during acid hydrolysis. The length distribution of the nanowhiskers is relatively broad, ranging from 100 to 400 nm, and the width varies from 5 to 35 nm.

**Fig. 2:**
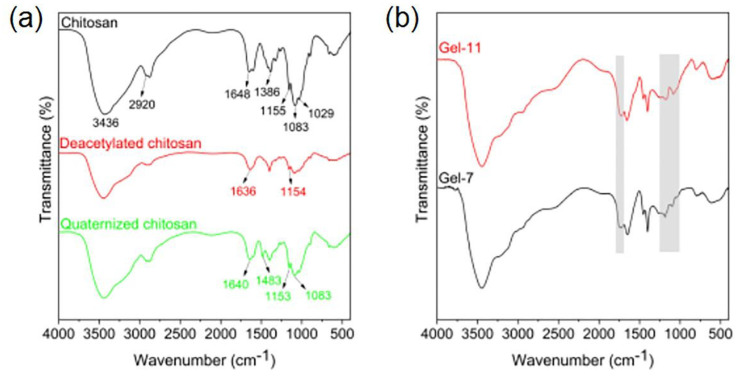
(a) FT-IR spectra of chitosan, deacetylated chitosan and quaternized chitosan; (b) FT-IR spectra of the hydrogel samples gel-7 and gel-11.

Quaternized chitosan-based nanocomposite hydrogels are synthesized according to the description of Scheme 1. First, quaternized chitosan (QCS) was synthesized by the reaction between deacetylated chitosan and 2,3-epoxypropyltrimethyl ammonium chloride (Scheme 1(b)). For the FT-IR spectrum of chitosan (Fig. 2(a)), there present several absorption peaks at 3436 cm^−1^ (O–H stretching overlapping with N–H stretching of primary amines), 2920 cm^−1^ (symmetric C–H stretching vibration of CH_2_ groups), 1648 cm^−1^ (C=O stretching vibration of the amide bond), 1083 cm^−1^ (stretching overlap of C6–O and C=O), 1029 cm^−1^ (stretching vibration of the C–O–C bridge bond), 1386 cm^−1^ (asymmetric C–H bending of CH_2_ groups), and 1155 cm^−1^ (C3–O stretching vibration).^43^ Compared with chitosan, the deacetylated chitosan presents a considerably broader absorption peak at 3400–3500 cm^−1^. This indicates an increased overlap of O–H and N–H stretching vibrations, corresponding to more amino groups due to acetyl group removal.^43^ Furthermore, the intensity of the C=O stretching vibration peak attributed to the amide bond at 1636 cm^−1^ decreases, indicating a reduction in the presence of acetyl groups. The asymmetric stretching peak of the bridge oxygen C–O bond at 1154 cm^−1^ also weakens, probably due to partial depolymerization during the deacetylation process.^44^ These results indicate that the chitosan was further deacetylated. FT-IR spectrum of QCS is similar to deacetylated chitosan. Notably, a new peak appears at 1483 cm^−1^ assigned to the C–H bending for the methyl group of the ammonium group in QCS.^45,46^ Additionally, the peaks at 1153 and 1083 cm^−1^, associated with C–O stretching in the glycosidic bridge, remain unchanged. These results suggest that the introduction of quaternary ammonium groups mainly occurs at the –NH_2_ sites.^47–51^ So, QCS was synthesized successfully.

The quaternized chitosan-based nanocomposite hydrogels were synthesized by the free radical polymerization and cooling process (Scheme 1(c)). Firstly, in the presence of QCS, ChWs and Zn^2+^, acrylamide (AM), acrylic acid (AA), and *N*,*N′*-methylene bisacrylamide (Bis) form the first network based on the poly(AM-*co*-AA) chain through a radical polymerization reaction. Furthermore, the Zn^2+^ forms the metal coordination bond with the –COO^−^ dissociated by COOH groups in the poly(AM-*co*-AA) chain. During the subsequent cooling process, a second network is formed through hydrogen bonding, ion interaction, and segment entanglement between QCS and the first network. Meanwhile, ChWs are uniformly distributed in the hydrogel network through hydrogen bonding with the polymer chains (Scheme 1(c)). Quaternized chitosan-based nanocomposite hydrogels with dual-network structures were constructed. For Gel-7, the peak around 1700 cm^−1^ is due to the C=O stretching vibration. The peaks at 1000–1250 cm^−1^ are related to C–O and C–N stretching vibrations (Fig. 2(b)). This confirms that radical polymerization between AA, AM, and Bis has successfully occurred. It can also be observed that the sample with QCS (Gel-11) exhibits a similar FT-IR spectrum to the sample without QCS (Gel-7), which is mainly because QCS and the first network are combined through non-covalent interactions.

### 3.2 Swelling properties and surface morphology of quaternized chitosan-based nanocomposite hydrogels

**Fig. 3:**
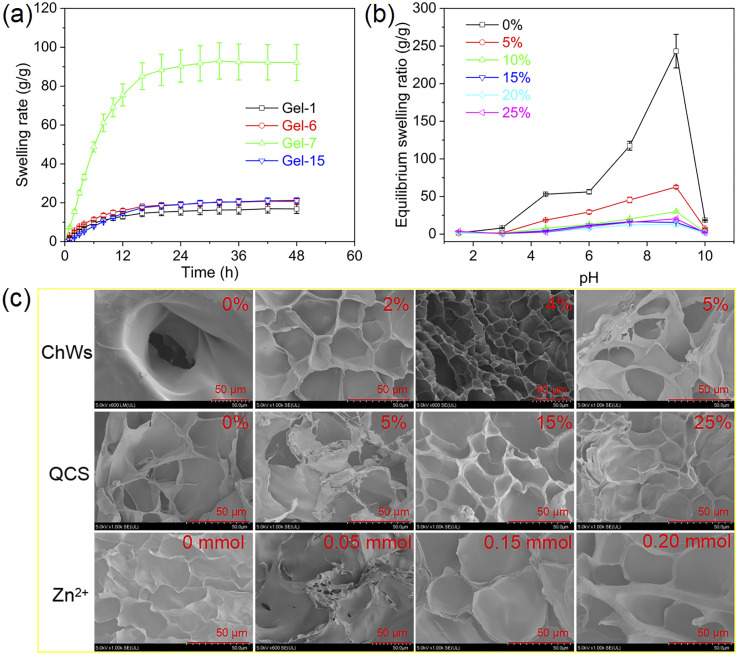
(a) Swelling behavior of quaternized chitosan-based nanocomposite dual-network hydrogels in buffer solution (pH = 7.4, *I* = 0.05); (b) equilibrium swelling ratios of the samples with various QCS contents at different buffer solutions; (c) SEM images of quaternized chitosan-based nanocomposite dual-network hydrogels.

The swelling behavior of quaternized chitosan-based nanocomposite hydrogels is displayed in Fig. 3(a). During the initial stage (<12 h), the swelling rate of the hydrogels increases rapidly. With increasing time, the swelling rate of the hydrogels increases slowly and eventually reaches equilibrium. Equilibrium swelling ratios of the samples at different buffer solutions are shown in Fig. 3(b) and S2. Except for the hydrogel without QCS, the equilibrium swelling ratio of other hydrogels at pH 1.5 is conspicuously larger than that at pH 3.0. This can be elucidated by the fact that at pH 1.5, the positive charges on the quaternary ammonium groups in QCS increase the electrostatic repulsion interaction within the hydrogel network, which promotes the swelling of the hydrogel. However, as the pH ascends to approximately 3.0, the –COOH groups start to partially dissociate into negatively charged –COO^−^, which electrostatically interacts with the positively charged quaternary ammonium groups, resulting in a decrease in electrostatic repulsion force within the hydrogel network. With the further increase of the pH value, the equilibrium swelling ratio of the hydrogels rises significantly, and all hydrogels exhibit the maximum equilibrium swelling ratio at pH 9.0. This is because the hydrogen bond interaction within the gel network is weakened, and a large number of carboxyl groups dissociate into –COO^−^ groups, which significantly increases the repulsive force within the hydrogel network, and consequently increases the equilibrium swelling ratio of the hydrogel.^52,53^ Nevertheless, at higher pH (>9.0), the equilibrium swelling ratios of all samples drop rapidly. The possible reason is that Na^+^ is absorbed and can form electrostatic attraction with the carboxyl anions on the side chains of the poly(AM-*co*-AA) chains, which may lead to a reduction in the electrostatic repulsive force within the gel network.^54,55^

The equilibrium swelling ratio of the sample with 1% of ChWs is larger than that of the sample without ChWs (Fig. S2(a)). This enhancement is primarily ascribed to the hydroxyl groups in chitin, which augment hydrophilicity and thereby strengthen the interactions between the hydrogel surface and water molecules, enhancing the water absorption capacity.^56^ The equilibrium swelling ratio of the hydrogels with lower ChWs content (1–3%) is larger than that of the hydrogels with higher ChWs content (≥4%). The higher ChWs content significantly increases the cross-linking density of the hydrogel network by promoting the formation of hydrogen bonding with QCS chain and poly(AM-*co*-AA) chains, attributable to the abundance of hydroxyl groups on the ChWs surface. This leads to restricted diffusion of water molecules within the network, thus resulting in a reduced swelling ratio. A previous study carried out by Spagnol *et al.*^56^ has also demonstrated that at excessively high concentrations, the swelling capacity decreases.

Compared to the sample containing QCS, the sample without QCS exhibits larger equilibrium swelling ratios at different buffer solutions (Fig. 3(b)). With the incorporation of QCS, the equilibrium swelling ratio of the hydrogel notably decreases. At higher QCS content (>10%), the equilibrium swelling ratio of hydrogel samples is almost the same in the same buffer solution. This trend might be ascribed to the increased crosslinking density caused by the introduction of QCS. Similarly, Wang *et al.* disclosed that all hydrogels containing QCS had lower swelling ratios than those without QCS, mainly because of the formation of a more tightly crosslinked network structure.^57^ Compared with the hydrogel sample without Zn^2+^, the sample containing 0.05 mmol of Zn^2+^ exhibits a remarkable increase in the equilibrium swelling ratio (Fig. S2(b)). Although the incorporation of Zn^2+^ forms the coordination bond with –COO^−^ groups, it also disrupts the existing hydrogen bonds within the network.^58^ When the Zn^2+^ content rises to 0.10 mmol, the hydrogel exhibits low equilibrium swelling ratios in different buffer solutions. With increasing of the Zn^2+^ content, the equilibrium swelling ratio increases. Zn^2+^ can form metal coordination bonds with –COO^−^ groups to increase the cross-linking density inside the hydrogel, leading to a decrease in the equilibrium swelling ratio.^59,60^ However, excess Zn^2+^ may exist in a free state or form monodentate coordination with –COO^−^ groups, which are not effective crosslinking points.^61^ Therefore, the higher Zn^2+^ content leads to an increased equilibrium swelling ratio.

SEM images of quaternized chitosan-based nanocomposite hydrogels are shown in Fig. 3(c). For the sample without ChWs, there is an obvious pore structure. With the increase of ChWs contents, the pore structure became more compact and uniform. The pore appeared more interconnected. This is because ChWs could form hydrogen bonds with poly(AM-*co*-AA) networks and QCS chains, which increases the crosslinking density of the hydrogels. The sample without QCS has porous channels that penetrate each other. With an increase in QCS content, the pore channels decrease, eventually disappear, and the surface becomes smoother. This is because the increase of QCS content not only increases the non-covalent interaction within the hydrogel network but also increases the entanglement between the QCS chain and the poly(AM-*co*-AA) network. This increases the crosslinking density of the hydrogel, resulting in fewer pore channels. This is consistent with the results of swelling experiments. Gong *et al.*^62^ also reported a similar phenomenon in the QCS/sodium alginate composite hydrogels. The hydrogel without Zn^2+^ shows a uniform porous structure, while the hydrogels containing Zn^2+^ display a relatively loose porous structure with irregular and sparsely distributed pores. The pore walls appeared thicker. This might result from the crosslinking effect caused by Zn^2+^, which influences microstructure. Uyanga *et al.*^63^ also reported that the incorporation of Zn^2+^ affects the surface roughness and porosity of carboxymethyl cellulose-based hydrogels while simultaneously altering their stability.

### 3.3 Mechanical properties and rheological behavior of quaternized chitosan-based nanocomposite hydrogels

**Fig. 4:**
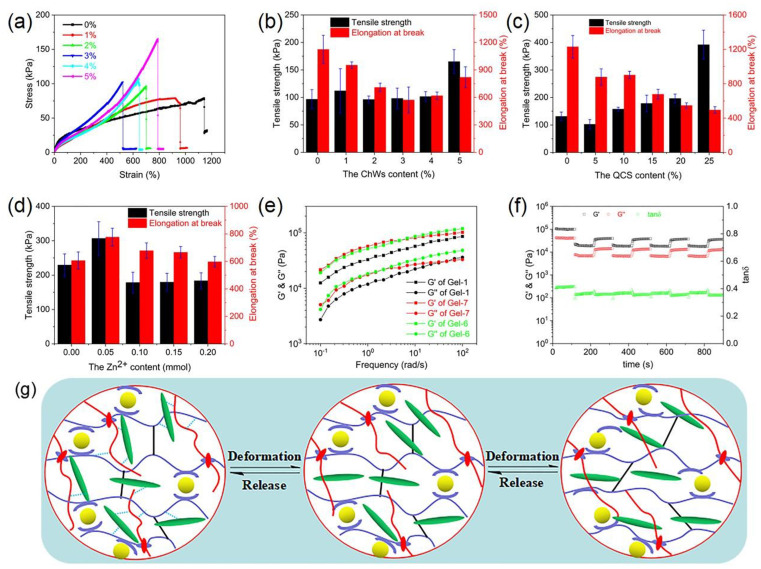
(a) Typical tensile stress–strain curves of quaternized chitosan-based nanocomposite dual-network hydrogels with various ChWs contents; (b–d) tensile strength and elongation at break ((b) the samples with various ChWs contents; (c) the samples with various QCS contents; (d) the samples with various Zn^2+^ contents); (e) oscillatory frequency sweeps of gel-1, gle-6 and gel-7; (f) cyclic continuous step strain measurements in which the strain was switched from 1% strain for 100 s to 100% strain for 100 s; (g) toughening and self-recovery mechanism of quaternized chitosan-based nanocomposite hydrogels.

The synthetic quaternized chitosan-based nanocomposite hydrogels can undergo significant deformation (Fig. S3), indicating that they have good mechanical properties. To quantitatively evaluate their mechanical performance, the hydrogel samples were tested through the stretching mode, and the obtained results are shown in Fig. 4(a) and S4. The tensile stress of all samples increases with the increase of tensile strain, showing good ductility. For the sample without ChWs, its tensile strength and elongation at break are 96.6 kPa and 1124%, respectively (Fig. 4(b)). As the ChWs content increases, the elongation at break of the hydrogel first decreases and then increases, and the sample with 3% of ChWs has a minimum elongation at break of 572%. Concerning tensile strength, at lower ChWs content (≤4%), there are no significant changes compared with the sample without ChWs. Intriguingly, the tensile strength of the sample with 5% of ChWs significantly rises to 165.1 kPa, marking an obvious enhancement compared to the sample without ChWs. This can be attributed to the fact that at higher ChWs content, the ChWs can establish connections (or interactions) with the hydrogel network *via* at least two or more crosslinking points, thereby withstanding mechanical stress and prominently enhancing the mechanical properties of the hydrogel.^23^ However, at lower ChWs content, the ChWs don't form effective crosslinking connections, thus restricting their reinforcing effect.^30^ Pereira *et al.*^30^ also found that a significant crosslinking effect and mechanical enhancement are witnessed only in nanocomposites containing at least 5 wt% ChWs.

Fig. 4(c) shows the tensile strength and elongation at break of hydrogel samples with various QCS contents. For the sample without QCS, its tensile strength and elongation at break are 131.5 kPa and 1231%, respectively. Tensile strength and elongation at break of the sample with 5% of QCS undergo a slight reduction than the sample without QCS. Nevertheless, as the QCS content increases, the tensile strength gradually increases, while the elongation at break decreases. The tensile strength of the sample with 25% of QCS could reach 391.9 kPa, and it has an elongation at break of 495%. At lower QCS content, there might be insufficient crosslinking to form an optimal network structure, resulting in a looser network. At higher QCS content, the increase of hydrogen bonds, ionic interactions, and the intertwining of polymer chains in the gel network leads to the increase of cross-link density of the hydrogel. This results in an increase in tensile strength and a decrease in elongation at break of the hydrogel.

Tensile strength and elongation at break of the hydrogel without Zn^2+^ are 229.2 kPa and 606%, respectively (Fig. 4(d)). With the addition of Zn^2+^, both the tensile strength and elongation at break of the sample with 0.05 mmol of Zn^2+^ increase significantly, attaining 306.8 kPa and 777%, respectively. However, at higher Zn^2+^ content (>0.05 mmol), their tensile strengths and elongation at breaks are 178.5–183.6 kPa and 598–678%, which don't change significantly with the increase of Zn^2+^. At lower Zn^2+^ content, the Zn^2+^ could facilitate an increase in crosslinking density within the hydrogel,^60^ thereby enhancing its mechanical strength and extensibility. However, the higher Zn^2+^ content may result in monodentate coordination with the ligand, which is an ineffective crosslinking point, so the hydrogel with higher Zn^2+^ content exhibits poor mechanical properties.

The frequency sweep of quaternized chitosan-based nanocomposite hydrogels is shown in Fig. 4(e). It is clear that the *G*′ values of the hydrogels consistently exceed its *G*′′ value, indicating a dominant elastic character throughout the frequency sweep. Compared to the sample Gel-1 without ChWs and the sample Gel-7 without QCS, the sample Gel-6 exhibits higher *G*′ and *G*′′ values at higher frequency range, which may be attributed to the dual network nanocomposite structure of the synthesized hydrogel. When the strain of 1% is applied, the *G*′ and *G*′′ of the sample Gel-6 are ∼100 kPa and ∼41 kPa, respectively (Fig. 4(f)). When the strain is increased to 100%, the *G*′and *G*′′ of the hydrogel rapidly decrease. However, when the strain is reduced to 1%, the *G*′ and *G*′′ of the hydrogel recover rapidly but slightly below the initial value. When the above process is repeated, both *G*′ and *G*′′ of the hydrogel can be recovered. The hydrogels have lower tan*δ* (0.3–0.4). When the applied larger strain at each cycle increases, the corresponding *G*′ and *G*′′ decreases (Fig. S5) and the tan*δ* of the hydrogel increases as the applied larger strain at each cycle increases, revealing that part of the three-dimensional network structure of the hydrogel is destroyed. It can be observed that when the large strain is reduced to 1%, the *G*′ and *G*′′ of the hydrogel could also recover. These results show that quaternized chitosan-based nanocomposite hydrogels have good self-recovery performance.

The excellent mechanical and self-recovery ability of quaternized chitosan-based nanocomposite hydrogel is closely related to its double-network nanocomposite structure (Fig. 4(g)). When the hydrogel sample is subjected to external force, the hydrogen bond between QCS, ChWs, and polymer chain segments in the first network is destroyed, allowing the polymer chains to move and dissipate energy. As the applied force increases, the metal coordination bonds in the first network formed based on poly(AM-*co*-AA) chains break and dissipate energy as sacrificial bonds. When the external force is removed, the covalent crosslinking points in the first network drive the polymer chain to quickly recover to its initial state, while the temporarily dissociated hydrogen and metal coordination bonds can be quickly reconstructed by reconfiguration during relaxation without any external stimulus.^31,64^ These results give quaternized chitosan hydrogels good toughness and self-recovery properties.

### 3.4 Biocompatibility of quaternized chitosan-based nanocomposite hydrogels

**Fig. 5:**
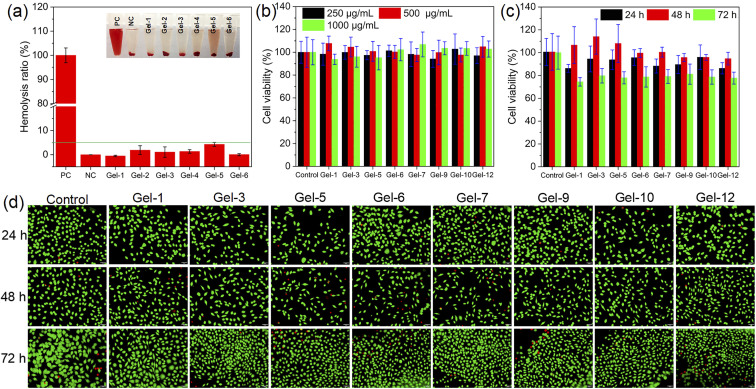
(a) Hemolysis ratio of quaternized chitosan-based nanocomposite dual-network hydrogels with various ChWs contents; (b) cell viability of L929 cells co-cultured for 24 h with extracts of the hydrogels at different concentrations; (c) cell viability of L929 cells co-cultured with the extract of the hydrogels at a concentration of 500 μg mL^−1^ for different times; (d) live/dead staining fluorescence images of L929 cells incubated with the hydrogel extract (500 μg mL^−1^) at different times.

The biocompatibility of quaternized chitosan-based nanocomposite hydrogels was evaluated by hemolysis test and cytotoxicity test, as shown in Fig. 5. Fig. 5(a) and S6 illustrate that the degree of hemolysis was the highest in the positive control group, while no hemolysis was observed in the negative control group. Except for sample Gel-12, no significant hemolysis was observed in the remaining samples. According to previous literature,^65^ the hemolysis ratio of the biomaterial is less than 5%, indicating that it has good blood compatibility. It is clear that except for sample Gel-12, the hemolysis ratio of the other samples was lower than 5%. However, the sample (Gel-12) without Zn^2+^ exhibits a significantly higher hemolysis ratio, which may be attributed to the role of Zn^2+^ in inhibiting hemolysis.^66–68^

The cytotoxicity of quaternized chitosan-based nanocomposite hydrogels was evaluated by MTT assay, as shown in Fig. 5(b) and (c). Fig. 5(b) shows that the cell viability of L929 cells for the nanocomposite hydrogels after co-culturing with the extract at different concentrations for 24 h is close to that of the control group. When the concentration of the extract is 1000 μg mL^−1^, the cell viability of L929 cells for Gel-1, Gel-3, Gel-5, Gel-6, Gel-7, Gel-9, Gel-10, and Gel-12 after co-culture with the extract for 24 h is 93.8%, 96.1%, 95.2%, 102.3%, 106.8%, 103.5%, 103.4% and 102.8%, respectively. Fig. 5(c) shows the effect of culture time on the viability of L929 cells. It can be observed that after 24 h of culture, it exhibits a lower cell viability. With the increase of culture time, the cell viability significantly increases, which is because L929 cells adapted to the extract environment with the increase in culture time. However, at 72 h of culture time, the cell viability decreases. This may be due to a decrease in cell proliferation and low DNA synthesis.^69^ The cell viability of L929 cells for Gel-1, Gel-3, Gel-5, Gel-6, Gel-7, Gel-9, Gel-10, and Gel-12 after co-cultured with the extract at a concentration of 500 μg mL^−1^ for 72 h is 74.4%, 79.8%, 77.9%, 78.7%, 79.2%, 81.2%, 78.7% and 77.7%, respectively. According to the evaluation criteria (GB/T 16886.5-2003 (ISO 10993-5: 1999)) of cytotoxicity of biomaterials,^70,71^ the material with cell viability larger than 75% can be considered non-cytotoxic. L929 cells were observed and evaluated by live/dead staining assay after co-culture with the hydrogel extract (Fig. 5(d)). After co-culturing with the hydrogel extract, there is a strong green fluorescence and only a very weak red fluorescence, which is similar to the control group. The result demonstrates again that the synthesized quaternized chitosan-based nanocomposite hydrogels are non-toxic, which is consistent with the result of the MTT assay. These results reveal that the synthesized quaternized chitosan-based nanocomposite hydrogels exhibit no cytotoxicity toward L929 cells, indicating their excellent biocompatibility.

### 3.5 Antibacterial properties of quaternized chitosan-based nanocomposite hydrogels

**Fig. 6:**
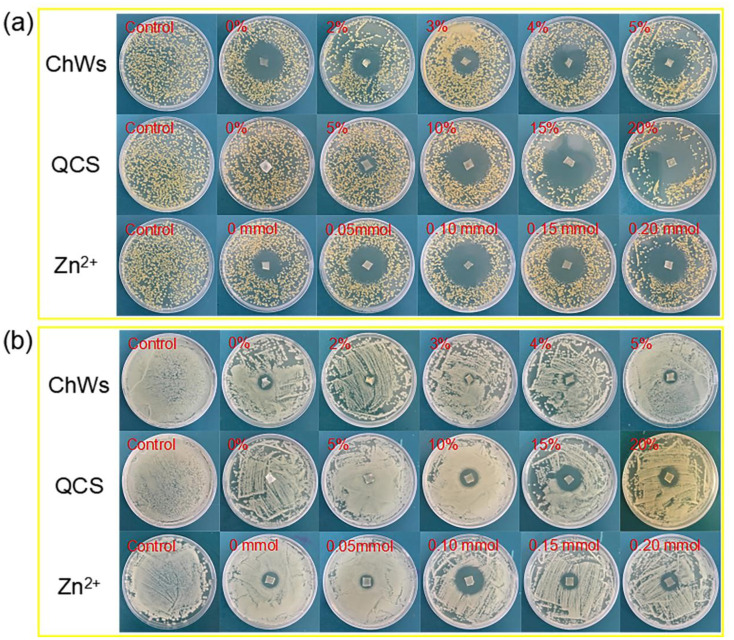
Results of the inhibition zone experiments of quaternized chitosan-based nanocomposite dual-network hydrogels against *S. aureus* (a) and *E. coli* (b).

The results of the inhibition zone experiments of quaternized chitosan-based nanocomposite hydrogels are shown in Fig. 6. Fig. 6 shows obvious inhibition zones around the samples with various ChWs contents and the samples with various Zn^2+^ contents in the medium inoculated with *S. aureus* and *E. coli.* However, no obvious inhibition zone is observed around the sample without QCS. With the increase of QCS content, the size of the inhibition zone around the samples increases significantly, indicating that the QCS content significantly affects the antibacterial efficacy of the hydrogel against *S. aureus* and *E. coli*. This indicates that the quaternized chitosan-based nanocomposite hydrogels have good antibacterial properties against both *S. aureus* and *E. coli*.

**Fig. 7:**
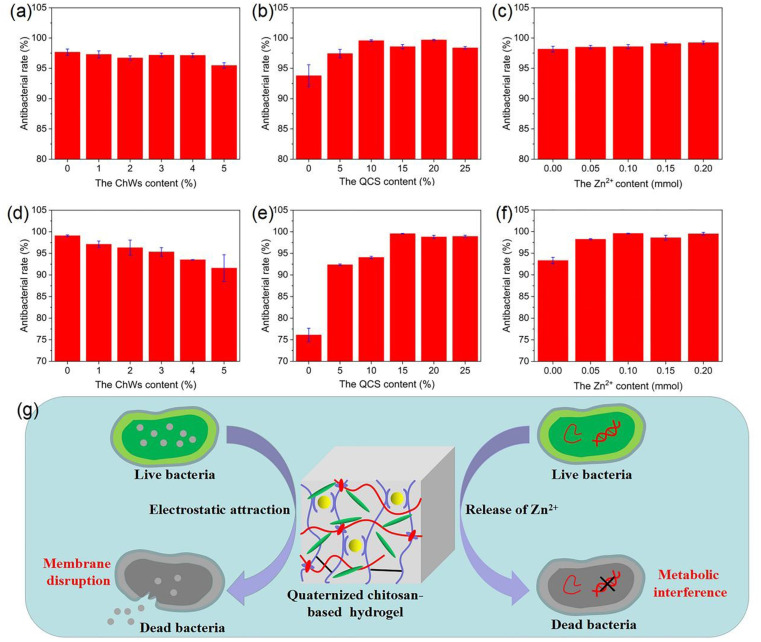
Antibacterial rates of quaternized chitosan-based nanocomposite hydrogels against *S. aureus* (a–c) and *E. coli* (d–f): (a and d) the samples with various ChWs contents; (b and e) the samples with various QCS contents; (c and f) the samples with various Zn^2+^ contents; (g) antibacterial mechanism of quaternized chitosan-based nanocomposite hydrogels.

On this basis, the antibacterial performance of quaternized chitosan-based nanocomposite hydrogel was quantitatively analyzed using the plate counting method, as shown in Fig. S7. It can be observed that the experimental group has a significant antibacterial effect on *S. aureus* and *E. coli* compared with the control group. The antibacterial rate acquired from the plate counting is shown in Fig. 7. For the sample without ChWs, its antibacterial rate against *S. aureus* and *E. coli* is 97.7% and 99.1%, respectively (Fig. 7(a) and (d)). With the increase of ChWs, the antibacterial rate of the hydrogel against *S. aureus* does not undergo significant changes (95.5∼97.3%). Conversely, a slight decrease in antibacterial rate against *E. coli* is observed as the ChWs increase. The antibacterial rate of the sample with 5% of ChWs against *E. coli* decreases to 91.6%*.* The possible reason is that the incorporation of ChWs leads to the formation of numerous hydrogen bonds in the hydrogel network, which could significantly increase the cross-linking points within the hydrogel. As a result, the swelling capacity of the hydrogel is reduced, which is detrimental to the thorough interaction between the antibacterial groups and ions within the hydrogel network and the bacterial solution. Fig. 7(b) and (e) show that the antibacterial rate of the sample without QCS against *S. aureus* and *E. coli* is 93.8% and 76.1%, respectively. With increasing QCS content, the antibacterial rate increases. When the QCS content is more than 15%, the hydrogel exhibited a higher antibacterial rate (>98%) for both bacterial strains. Similar phenomena have also been reported in the polyacrylamide/quaternary ammonium chitosan hydrogels.^72^ This is attributed to the positive charge of quaternary ammonium chitosan.

The hydrogel sample without Zn^2+^ exhibits a high antibacterial rate against *S. aureus* and *E. coli*, achieving 98.2% and 93.3%, respectively (Fig. 7(c) and (f)). With the introduction of Zn^2+^ content, the hydrogel's antibacterial rate increases significantly, which is attributed to the inherent antibacterial properties of Zn^2+^.^73^ The antibacterial mechanism involves electrostatic interactions between the positively charged Zn^2+^ and the negatively charged bacterial cell membranes. This interaction alters cell membrane permeability, leading to the leakage of cellular contents and subsequent cell death.^73^ For the sample with a Zn^2+^ content of 0.10 mmol, its antibacterial rate against *S. aureus* and *E. coli* reaches 98.6% and 99.6%, respectively. At higher Zn^2+^ content (>0.10 mmol), the sample against *S. aureus* and *E. coli* exhibits a high antibacterial rate, which is stable without significant changes with Zn^2+^ content. This is likely due to the majority of the bacteria being eliminated by the initial low contents of Zn^2+^, so the higher Zn^2+^ content does not further improve the antibacterial rate. These results indicate that the quaternized chitosan-based nanocomposite hydrogels have inherent antibacterial activity. As shown in Fig. 7(g), since the synthesized hydrogel contains positively charged groups, it can adsorb bacteria to the surface of the hydrogel through electrostatic attraction.^74^ This can damage the structure of the bacteria's cell membrane, causing material to flow out of the cell. Another possible reason is that Zn^2+^ released by the hydrogel penetrates the cell membrane and enters the cell interior, interfering with DNA replication and transcription processes.^75^ The antibacterial mechanism of a hydrogel may also be a synergistic effect of the two antibacterial mechanisms described above.

### 3.6 Blood coagulability of quaternized chitosan-based nanocomposite hydrogels

**Fig. 8:**
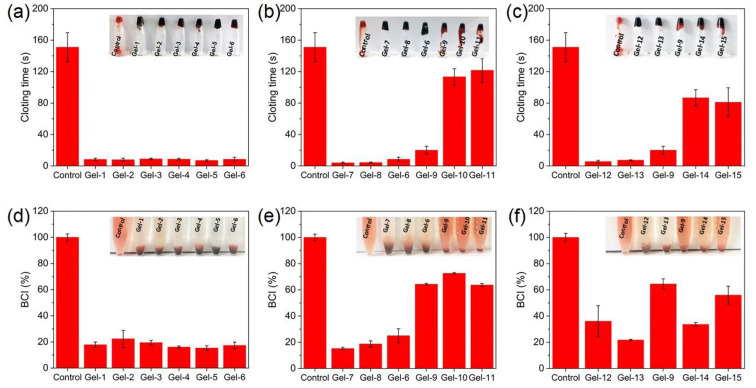
Whole blood coagulation time (a–c) and whole blood coagulation index (d–f) of quaternized chitosan-based nanocomposite dual-network hydrogels: (a and d) the samples with various ChWs contents; (b and e) the samples with various QCS contents; (c and f) the samples with the various Zn^2+^ contents.

Whole blood coagulation time (BCT) and whole blood coagulation index (BCI) were used to evaluate the blood coagulation performance of the synthesized hydrogels, as shown in Fig. 8. Fig. 8(a–c) shows that the centrifuge tubes are inverted for all samples at the corresponding coagulation time, and the blood showed a coagulation state, indicating that they had completed coagulation at the corresponding time. The BCT value of all samples is smaller than that of the control group (Fig. 8(a–c)). The shorter the coagulation time, the better the hemostatic effect of the material. So the samples exhibit rapid hemostasis ability. Fig. 8(a) shows that the BCT values of the samples with various ChWs contents are all less than 10 s. The sample Gel-7 without QCS also has a low BCT value (∼4 s) (Fig. 8(b)). With the increase of QCS content, the BCT value of the samples increases rapidly. This is attributed to the dependence of thrombin generation on a negatively charged microenvironment during the coagulation process.^76,77^ However, with the increased content of QCS, there is a notable rise in the density of positive charges on the hydrogel surface, which may disrupt the local negatively charged environment. Consequently, this disruption inhibits thrombin generation and delays the coagulation process. The effect of Zn^2+^ content on the BCT value was also measured (Fig. 8(c)). The BCT value of the sample Gel-12 without Zn^2+^ is only several seconds. The BCT value increases first and then slightly decreases as the Zn^2+^ content increases. The sample (Gel-14) with 0.15 mmol of Zn^2+^ exhibits a long coagulation time (∼86.7 s). This phenomenon is likely due to Zn^2+^ inhibiting the release of procoagulant substances from leukocytes in a concentration-dependent manner.^78^

The BCI values of the hydrogel samples are shown in Fig. 8(d–f). The smaller the BCI value, the better the hemostatic ability of the material.^79^Fig. 8(d–f) shows that the blood of the control group did not show an obvious coagulation phenomenon within 2 min, while the blood added with hydrogel samples showed different degrees of blood coagulation. In Fig. 8(d), the samples with various ChWs contents have lower BCI values (15.4%∼22.4%), showing excellent blood coagulation performance. This is consistent with the result of whole blood coagulation time. The BCI value of the sample Gel-7 without QCS is 15.2% (Fig. 8(e)). With increasing QCS content, the BCI value of the sample increases. For the samples (Gel-9, Gel-10 and Gel-11) with higher QCS content (15%∼25%), their BCI values are 63.7%∼72.6%. This indicates that increasing the content of QCS results in a deterioration of the blood coagulability of quaternized chitosan-based nanocomposite hydrogels, which has been confirmed by the tests of whole blood coagulation time. The BCI value (21.7%∼64.5%) of the sample has no significant correlation with the change of Zn^2+^ content (Fig. 8(f)), and it is significantly lower than that of the control group. These results indicate that quaternized chitosan-based nanocomposite hydrogels have fast and excellent blood coagulation properties, so the synthesized hydrogels are expected to be used as hemostatic materials.

## 4. Conclusions

Quaternized chitosan-based nanocomposite hydrogels were synthesized by combining dual-network and nanocomposite technology. The synthesized hydrogels exhibited good pH sensitivity and a three-dimensional network structure. The mechanical properties and antibacterial properties of quaternized chitosan-based nanocomposite hydrogels can be effectively adjusted by controlling ChWs, QCS, and Zn^2+^ contents. The mechanical properties of the quaternized chitosan-based nanocomposite hydrogels were improved by increasing QCS content, and the sample with 25% QCS exhibits a tensile strength of 391.9 kPa and an elongation at break of 495%. The increased QCS and Zn^2+^ contents significantly improved the antibacterial property of the nanocomposite hydrogels, and the maximum antibacterial rate against *E. coli* and *S. aureus* could reach 99%. The quaternized chitosan-based nanocomposite hydrogels also had good biocompatibility and rapid hemostatic ability. This study provides a new strategy to synthesize hydrogels with excellent mechanical properties, antibacterial properties, and rapid hemostatic properties. These features make the hydrogel a promising candidate for practical biomedical applications, including wound management, hemostatic materials, and infection control.

## Conflicts of interest

There are no conflicts to declare.

## Supplementary Material

RA-015-D5RA03440J-s001

## Data Availability

All data supporting the findings of this study are available within the article and its SI. Supplementary information is available. See DOI: https://doi.org/10.1039/d5ra03440j.
