# Supplementary material for: Biocompatible quaternized chitosan-based nanocomposite hydrogels with antibacterial and rapid hemostatic properties
Source: RSC Adv. 2025 Aug 26;15(37):30202–16. doi: 10.1039/d5ra03440j (PMC12380049; doi:10.1039/d5ra03440j)
Supplement: RA-015-D5RA03440J-s001 [file RA-015-D5RA03440J-s001.pdf]

Supplementary Information (SI) for RSC Advances.
This journal is © The Royal Society of Chemistry 2025

Biocompatible quaternized chitosan-based nanocomposite hydrogels with antibacterial and rapid hemostatic properties

Juanni Zhang [a], Farhan Mohd Said [a] *, Ruixue Lv [b], Nur Fathin Shamirah Daud [a], Zhanxin Jing [b]

[a] Faculty of Chemical and Process Engineering Technology, Universiti Malaysia Pahang Al-Sultan Abdullah, Lebuh Persiaran Tun Khalil Yaakob, 26300 Kuantan, Pahang, Malaysia.

[b] College of Chemistry and Environment, Guangdong Ocean University, 524088 Zhanjiang, Guangdong, China.

Supporting Materials

**Corresponding author:* Farhan Mohd Said (farhan@umpsa.edu.my)

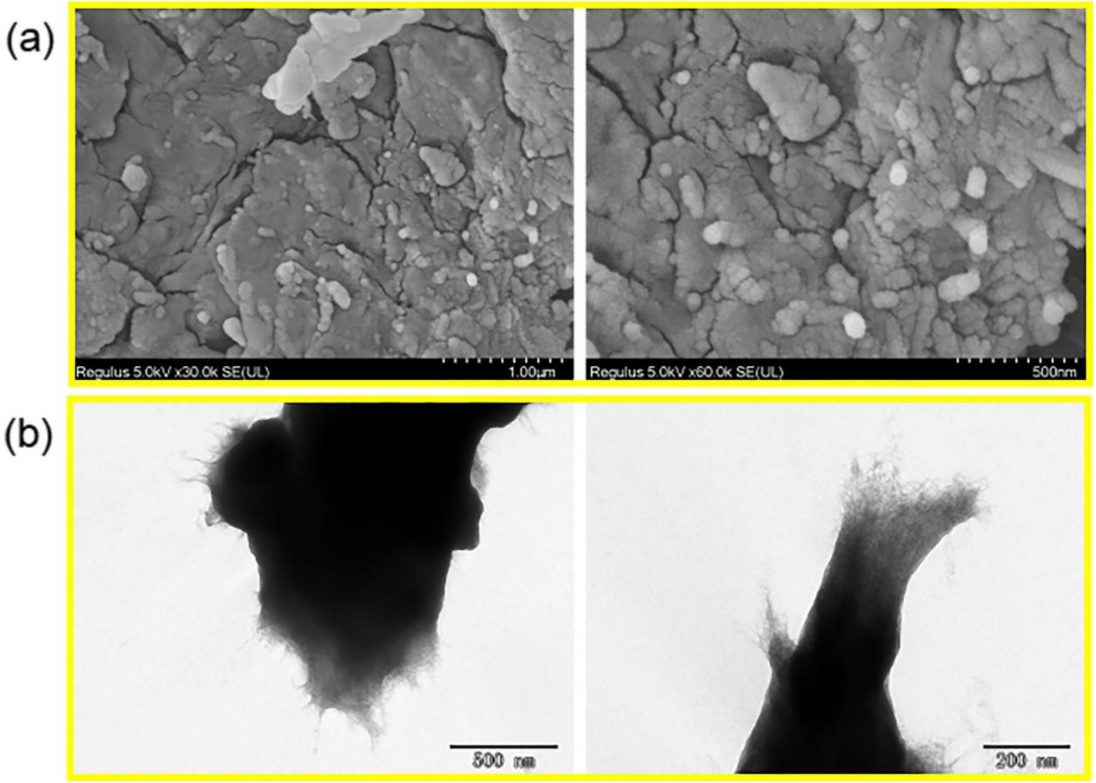

Figure S1 SEM images (a) and TEM images (b) of chitin

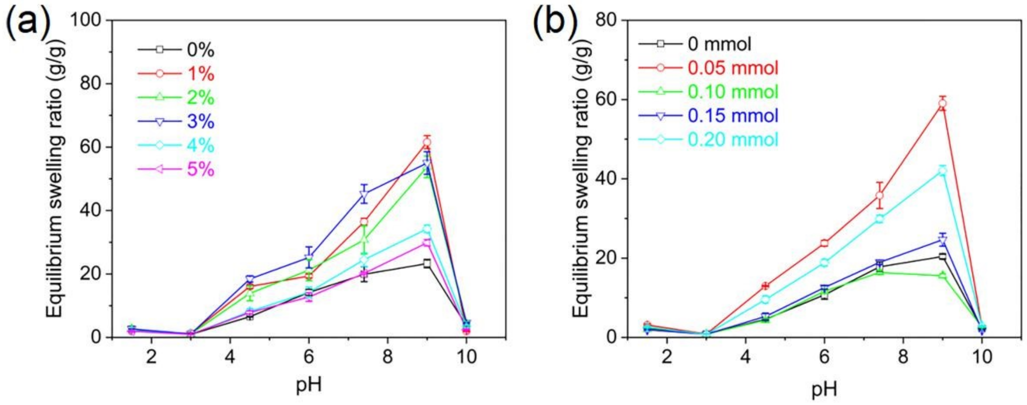

Figure S2 Equilibrium swelling ratios of quaternized chitosan-based nanocomposite dual-network hydrogels at different buffer solutions: (a) the samples with various ChWs contents; (b) the samples with various Zn^{2+} contents

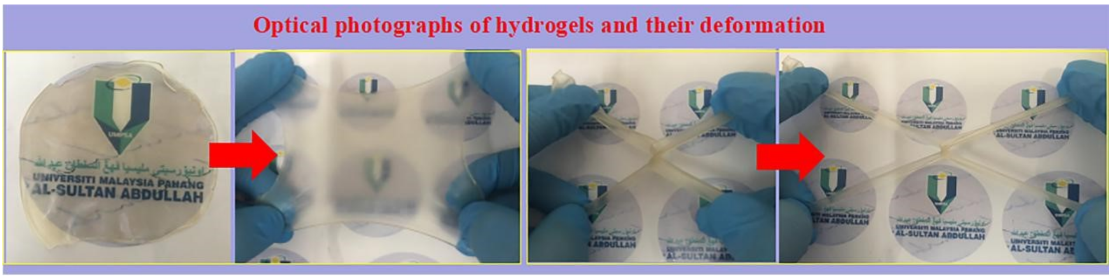

Figure S3 Optical photographs of quaternized chitosan-based nanocomposite hydrogels and their different deformations

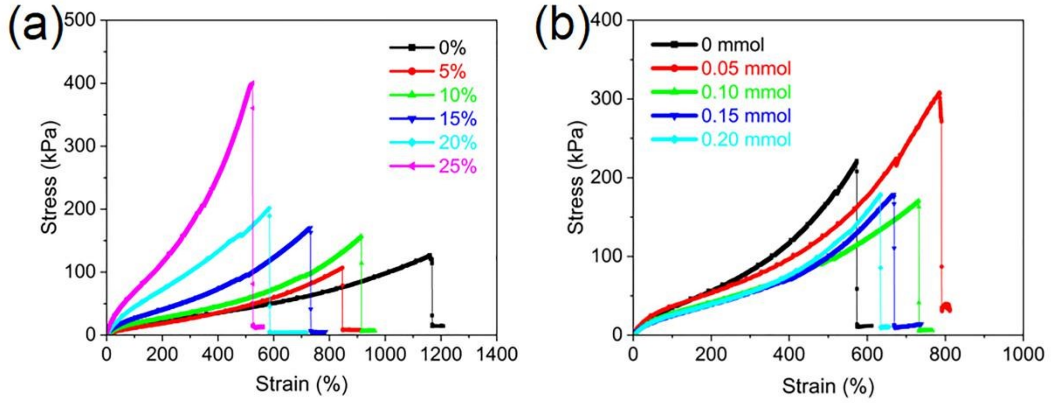

Figure S4 Typical tensile stress-strain curves of quaternized chitosan-based nanocomposite dual-network hydrogels: (a) the samples with various QCS contents; (b) the samples with various Zn^{2+} contents

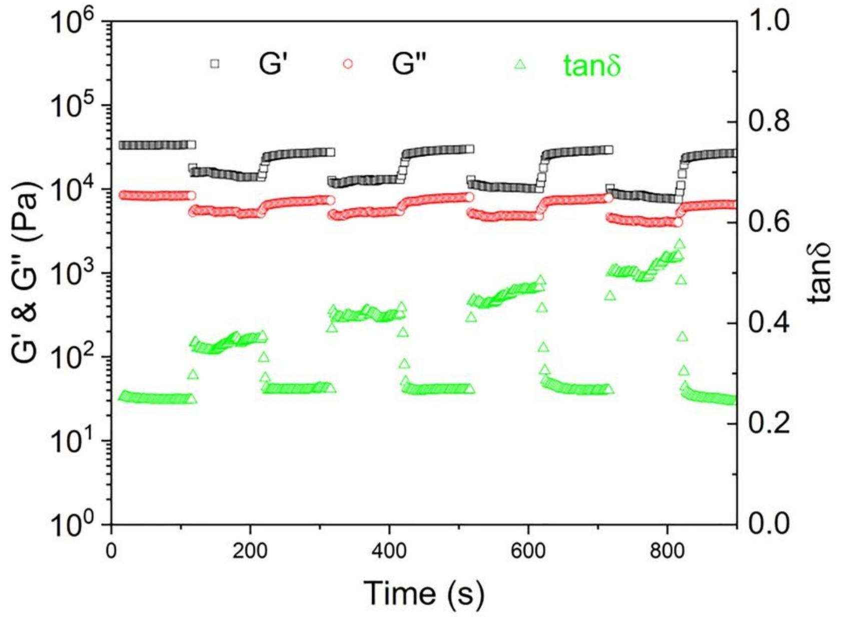

Figure S5 Cyclic continuous step strain measurements in which the strain was switched from 1% strain for 100 s to various larger strains (100%, 200%, 300% and 400%) for 100 s

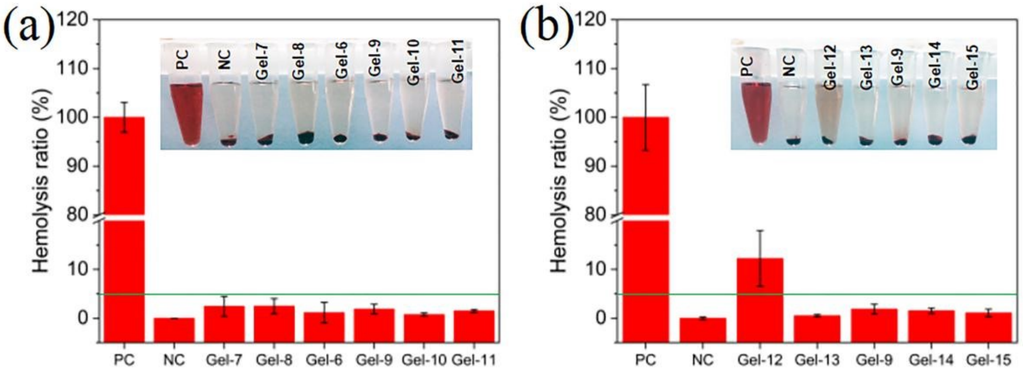

Figure S6 Hemolysis ratio of quaternized chitosan-based nanocomposite dual-network hydrogels: (a) the samples with various QCS contents; (b) the samples with various Zn^{2+} contents

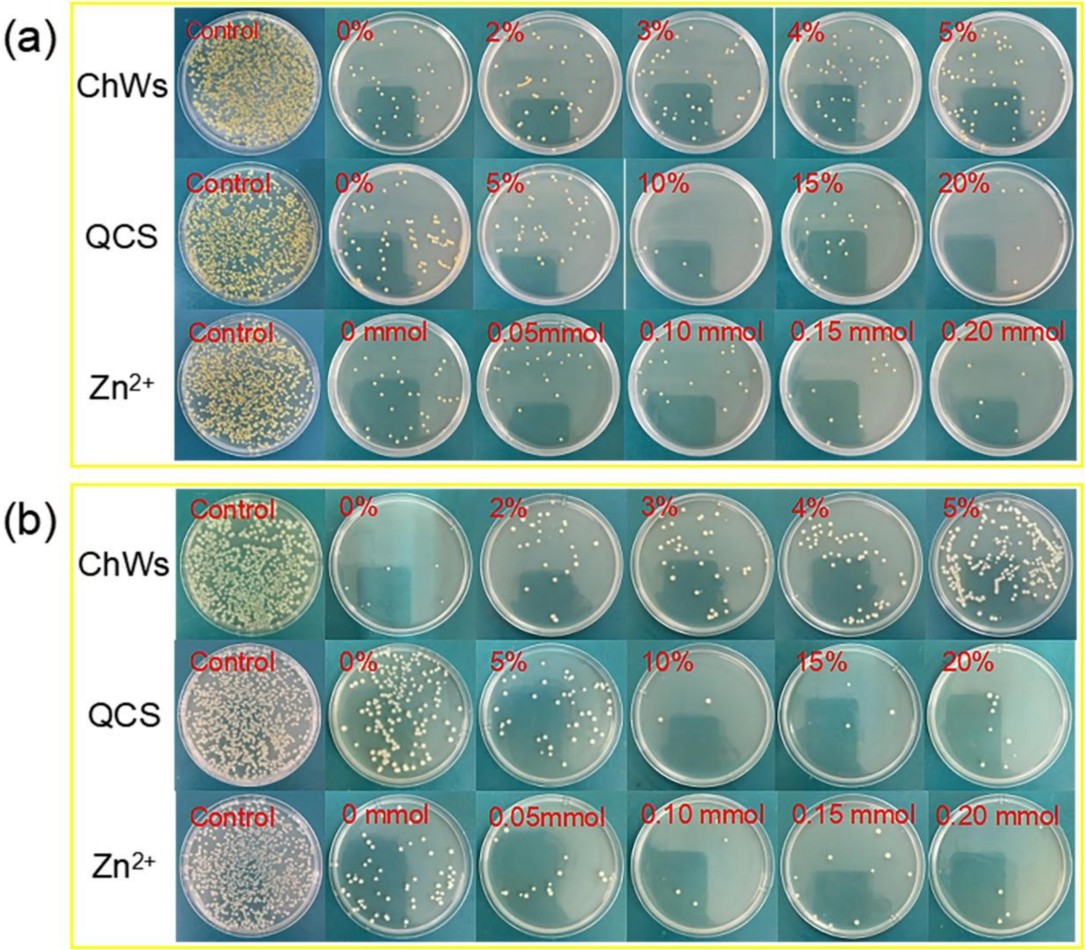

Figure S7 Optical photographs of the results of plate count experiments on the antibacterial properties of hydrogels against *S. aureus* (a) and *E. coli* (b)
